# Health professionals’ and service users’ perspectives of shared care for monitoring wet age-related macular degeneration: a qualitative study alongside the ECHoES trial

**DOI:** 10.1136/bmjopen-2014-007400

**Published:** 2015-04-21

**Authors:** D Townsend, B C Reeves, J Taylor, U Chakravarthy, D O'Reilly, R E Hogg, N Mills

**Affiliations:** 1 School of Social and Community Medicine, University of Bristol, Bristol, UK; 2 School of Clinical Sciences, University of Bristol, Bristol Royal Infirmary, Bristol, UK; 3 Centre for Experimental Medicine, Institute of Clinical Science, Queen's University Belfast, Belfast, UK; 4 Centre for Public Health, Institute of Clinical Sciences, Queens University Belfast, Belfast, UK

**Keywords:** OPHTHALMOLOGY, QUALITATIVE RESEARCH

## Abstract

**Objectives:**

To explore the views of eye health professionals and service users on shared community and hospital care for wet or neovascular age-related macular degeneration (nAMD).

**Method:**

Using maximum variation sampling, 5 focus groups and 10 interviews were conducted with 23 service users and 24 eye health professionals from across the UK (consisting of 8 optometrists, 6 ophthalmologists, 6 commissioners, 2 public health representatives and 2 clinical eye care advisors to local Clinical Commissioning Groups). Data were transcribed verbatim and analysed thematically using constant comparative techniques derived from grounded theory methodology.

**Results:**

The needs and preferences of those with nAMD appear to be at odds with the current service being provided. There was enthusiasm among health professionals and service users about the possibility of shared care for nAMD as it was felt to have the potential to relieve hospital eye service burden and represent a more patient-centred option, but there were a number of perceived barriers to implementation. Some service users and ophthalmologists voiced concerns about optometrist competency and the potential for delays with referrals to secondary care if stable nAMD became active again. The health professionals were divided as to whether shared care was financially more efficient than the current model of care. Specialist training for optometrists, under the supervision of ophthalmologists, was deemed to be the most effective method of training and was perceived to have the potential to improve the communication and trust that shared care would require.

**Conclusions:**

While shared care is perceived to represent a promising model of nAMD care, voiced concerns suggest that there would need to be greater collaboration between ophthalmology and optometry, in terms of interprofessional trust and communication.

**Trial registration number:**

ISRCTN07479761.

**Strengths and limitations of this study**

- This is the first study to explore perspectives of shared care for neovascular age-related macular degeneration, and provides important insights into possible barriers and facilitators to implementation that would need to be incorporated into the planning of any pilot scheme.
- A purposeful sampling approach was adopted to ensure that the feasibility and acceptability of the proposed shared model of care was captured from a range of perspectives, and the constant comparative method provided an opportunity to highlight similarities and differences between the participants.
- Optometrists and ophthalmologists were attending specialist conferences and therefore, may represent a more motivated and enthusiastic group of professionals. Similarly, service users were recruited from Macular Society support meetings; so they may represent a more proactive and informed group of patients.

## Introduction

Age-related macular degeneration (AMD) is one of the leading causes of severe visual loss in the world,^1^ accounting for over half of blind and partial sight certifications in the UK.^2^ In 2010, it was estimated that 608 213 people in the UK had AMD, with an expected increase to 755 867 by the end of the decade.^1^ This will continue to place an enormous strain on healthcare services in the UK, where the cost of sight impairment was estimated to be £22 billion in 2008.^3^ In addition, the vision loss associated with AMD has been shown to severely impact on a patient's quality of life, being associated with loss of independence, depression and social isolation.^4^

Since 2008, the National Institute for Health and Care Excellence (NICE) recommended that the National Health Service (NHS) hospital eye service (HES) should use the antivascular endothelial growth factor drug ranibizumab in England and Wales to treat patients with neovascular age-related macular degeneration (nAMD) until the neovascular process becomes quiescent or ‘stable’ for at least 3 months. The disease can reactivate at any time so patients with quiescent disease are required to be monitored by monthly visits to assess the need for retreatment.^5^ This has created a substantial increase in HES workload, with clinics struggling to provide regular monthly reviews.^6^ Timely retreatment has been shown to be critical in maintaining visual acuity and preventing vision loss. Since delays in follow-up beyond the recommended monthly interval can result in vision loss that may be irreversible and consequently impose additional burdens on health and social care, a number of potential solutions to relieve the HES burden have been outlined.^6^

One possible strategy to minimise the load on secondary care is the use of community optometrists for monitoring ‘stable’ patients (patients deemed to be at low risk of requiring retreatment). The ‘Effectiveness of Community versus Hospital Eye Service’ (ECHoES) trial was a web-based trial (ISRCTN: 07479761) designed to assess whether community-based optometrists can monitor patients with nAMD that has been rendered quiescent, using colour and optical coherence tomography (OCT) images and clinical examination, and accurately identify who needs to be referred back to an ophthalmologist for retreatment if the disease becomes active again. This would be a pre-requisite for establishing a shared care scheme whereby the greater part of the monitoring of patients with stable nAMD disease could be undertaken in a community setting. This change in service provision would free up clinic capacity in the HES and allow clinicians to concentrate on monitoring and treating patients during the ‘active’ phase of nAMD.

However, even if community optometrists can be shown to have levels of diagnostic accuracy and management competence equivalent to ophthalmologists, there are many other reasons why a change in service provision may be challenging. These include staff training and competency assessment, costs and patient acceptance.^6^ It is, therefore, important to ascertain the perspectives of health professionals and service users.^7^ Various studies have explored the acceptability and feasibility of enhancing optometric services to relieve the HES burden for a range of eye diseases;^8–12^ however, none have done so specifically for monitoring nAMD. Therefore, the purpose of the current study was to explore health professionals’ and service users’ perspectives of shared care for nAMD through focus groups and interviews, as part of the ECHoES trial.

## Method

### Recruitment and sampling

Optometrists and ophthalmologists were recruited from those due to attend annual UK-based specialist conferences—the National Optical Conference and the Royal College of Ophthalmologists annual congress. Those who expressed an interest after hearing about the study were emailed further information along with a participant information leaflet. Leads of Clinical Commissioning Groups (CCGs) in England were emailed study information and asked to forward it to the general practitioners (GPs)/Commissioners in each CCG who were involved in commissioning eye healthcare. In each case, those who were initially interested were asked to contact the researcher for more information. Additional clinical advisors and public health representatives were identified by the snowball technique, in which healthcare professionals provided the names of other potentially interested colleagues.

Service users with nAMD were recruited from local support groups organised by the Macular Society (a UK-based charity for anyone affected by central vision loss). Service users with any history of nAMD were invited to join the study (regardless of whether they had nAMD in one eye or both, dry AMD in their other eye, or were currently receiving or had any treatment in the past). Three support groups in South West England were purposefully selected–one based in a major city, a large town and a rural village. DT attended local support meetings to explain about the research and provide attendees with a participant information leaflet. Contact details of those potentially interested were obtained and they were telephoned a week later to discuss the study further.

A purposeful sampling strategy was used to ensure that the feasibility and acceptability of the proposed shared model of care for nAMD was captured from a range of perspectives. Within this sampling approach, maximum variation was sought in relation to profession, age, gender and geographic location (for health professionals), and gender, age, type of nAMD and time since diagnosis (for service users). Participant characteristics were assessed as the study progressed, and individuals or groups that were under-represented were targeted (ie, commissioners or clinical advisors). Where it was felt that variation had been achieved, potential participants were thanked for their interest and informed that sufficient numbers had been recruited (ie, optometrists).

### Data collection

Focus groups were conducted separately for optometrists and ophthalmologists at the specialist conferences. Focus groups with service users were held alongside the Macular Support local meetings in a separate room. Individual interviews were conducted with healthcare commissioners, clinical advisors to CCGs and public health representatives at a convenient location or over the telephone if preferred. Discussions were led by the participants themselves, with DT flexibly guiding focus groups and interviews by occasionally probing for more information, clarifying any ambiguous statements, encouraging the discussion to stay on track, and providing an opportunity for all participants in the focus groups to contribute to the discussion.

Separate topic guides were developed for service users, optometrists/ophthalmologists (with additional questions for each professional group), and all other health professionals to ensure that discussions within each group covered the same basic issues, but with sufficient flexibility to allow new issues of importance for the informants to emerge. These were based on the study aims, relevant literature and feedback from eye health professionals in the ECHoES study team, and consisted of open-ended questions about the current model of care for nAMD and perspectives of stable patients being monitored in the community by optometrists (see online supplementary appendix). These were adapted as the analysis progressed to enable exploration of emerging themes. Written consent was obtained from each participant at the start of the focus group or interview. A digital voice recorder was used to record the discussions. All participants were offered a £20 gift voucher to thank them for their time. The focus groups and interviews were conducted between November 2013 and June 2014.

### Data analysis

Audiorecordings were transcribed verbatim and checked against the audiorecording for accuracy. Transcripts were imported into NVivo (V.10), where data were systematically assigned codes and analysed using constant comparison methods derived from grounded theory methodology.^13^ Emerging themes were discussed with a second experienced social scientist (NM), with reference to the raw data. Data collection and analysis proceeded in parallel, with emerging findings informing further sampling and data collection. Analysis continued until the point of data saturation, that is, the point at which no new themes emerged.

## Results

### Participants

#### Health professionals

One focus group was undertaken with optometrists and another with ophthalmologists, lasting a mean of 91 min. Ten interviews were conducted with other health professionals (6 face-to-face and 4 over the telephone) with a mean duration of 48 min. In total, 24 health professionals were recruited (8 optometrists, 6 ophthalmologists, 2 public health representatives, 6 NHS commissioners and 2 clinical eye care advisors to their local CCGs). Of these, 12 were women and 12 men. These participants had a mean age of 39 years (range 31–64 years) and had been in their profession for a mean of 21 years (range 4–40 years). Although none had participated in any form of shared care for nAMD, 11 had experience of shared care schemes for other conditions (eg, diabetic retinopathy screening and ocular hypertension monitoring). Table 1 provides background information on the health professional participants. Years in profession and location are not given for the ‘other’ health professionals (ie, those other than ophthalmologists and optometrists) to protect their anonymity given their unique roles. However, these other professional participants were distributed geographically throughout England and they had been in their profession for an average of 20 years (range 8–37 years).

**Table 1:** Health professional participants’ background

Participant*	Role(s)	Years in profession†	Location†
Focus group 1			
Optom1	Optometrist	37	South West England
Optom2	Optometrist	28	South East England
Optom3	Optometrist	29	North West England
Optom4	Optometrist	37	West Midlands
Optom5	Optometrist	15	South West England
Optom6	Optometrist	20	South West England
Optom7	Optometrist	36	South West England
Optom8	Optometrist	40	West Midlands
Focus group 2			
Ophthalm1	Ophthalmologist	20	North West England
Ophthalm2	Ophthalmologist	20	London
Ophthalm3	Ophthalmologist	4	Dundee
Ophthalm4	Ophthalmologist	7	East Midlands
Ophthalm5	Ophthalmologist	17	North West England
Ophthalm6	Ophthalmologist	3	South West England
Interviews			
CA1	Optometrist, clinical advisor to local CCG	–	–
CA2	Optometrist, clinical advisor to local CCG	–	–
Comm1	Commissioner, pharmacist	–	–
Comm2	Commissioner, GP	–	–
Comm3	Commissioner, GP	–	–
Comm4	Commissioner, GP	–	–
Comm5	Commissioner, pharmacist	–	–
Comm6	Commissioner, optometrist	–	–
PH1	Member of Eye Health Local Professional Network, optometrist	–	–
PH2	Optical advisor for eye charity, optometrist	–	–

*Health professionals are identified by their professional role and their individual participant number.

†Owing to their unique role, the other health professionals’ years in profession and location have been removed to protect anonymity.

CA, clinical advisor; Comm, Commissioner; CCG, Clinical Commissioning Group; GP, general practitioner; Ophthalm, ophthalmologist; Optom, optometrist; PH, public health representative.

#### Service users

Three focus groups were conducted with 23 service users (with 7 or 8 in each group) and lasted a mean of 71 min. The sample consisted of 15 women (65%) and 8 men (35%) who described themselves as white British. The sampling strategy intended to recruit individuals from a mix of ethnicities, but all those at the supporting groups who were willing to be contacted were white British. They had a mean age of 82 years (range 72–93 years). All had nAMD, attended the same eye hospital in a major city and had been diagnosed, on average, 5.9 years ago (range 6 months to 20 years). Nine participants had active nAMD in one eye (39%), nine had quiescent nAMD in one eye (39%), four people had active nAMD in both eyes (18%) and one person had quiescent nAMD in both eyes (4%). Eight participants had dry AMD in their other eye (35%). Table 2 provides service user participants’ demographic and health-related details.

**Table 2:** Service user participants’ demographic and health-related details

Participant*	Gender	Age	Location of support group	Information about condition	Time since diagnosis (years)
Focus group 3					
Arthur	Male	80	City	Advanced active nAMD in one eye	20
Edith	Female	83	City	Inactive nAMD in one eye	7
Edward	Male	78	City	Active wet in one eye, dry in other	10
Elizabeth	Female	81	City	Inactive wet in one eye, dry in other	12
Harriett	Female	86	City	Inactive nAMD in one eye, dry in other	2.5
Kath	Female	77	City	Inactive in nAMD in both eyes	15
Ruth	Female	87	City	Active nAMD in one eye	10
Tom	Male	79	City	Inactive wet in one eye, dry in other	13
Focus group 4					
Alice	Female	79	Large town	Inactive nAMD in one eye	5
Debbie	Female	84	Large town	Advanced active nAMD in one eye, dry in other	4
Julie	Female	86	Large town	Active nAMD in one eye, dry in other	6
Mandy	Male	93	Large town	Active nAMD in one eye	6
Maria	Female	93	Large town	Inactive nAMD in one eye	20
Pam	Female	83	Large town	Active nAMD in one eye, dry in other	4
Ralph	Male	79	Large town	Active nAMD in one eye	7
Robert	Female	78	Large town	Active nAMD in both eyes	10
Focus group 5					
George	Male	85	Rural village	Active nAMD in both eyes	6
Harry	Male	72	Rural village	Inactive nAMD in one eye	1
Henry	Male	82	Rural village	Active nAMD in one eye, dry in other	4
Olivia	Female	84	Rural village	Active nAMD in both eyes	8
Pat	Female	78	Rural village	Active nAMD in both eyes	7
Tracey	Female	77	Rural village	Inactive nAMD in one eye	11
Yvonne	Female	76	Rural village	Inactive nAMD in one eye	1

*Pseudonyms were assigned to participants.

nAMD, neovascular age-related macular degeneration.

### Analysis

Six main themes emerged from the data, as detailed below.

#### Current clinic capacity: pushed to the limit

Many health professionals stated that the number of repeat hospital visits for patients was rising exponentially, which they attributed to an increase in the number of patients who were being diagnosed with nAMD and new government guidelines for treatment. Hospital clinics were felt to be ‘pushed to their limit’ (Optom7, Ophthalm3) and the ophthalmologists felt frustrated that their time was mostly spent on stable patients who did not require treatment.

You are taking the potential time from the ones that actually need the care. There is a limit of work that you can do. I mean you can't go home at 8 every day. (Ophthalm2)

Both the health professionals and service users described how patients would often have to wait for long periods of time for their appointment because of how busy the eye hospital clinics were.

Well, quite honestly when you go to the eye hospital, it always seems to be packed out left, right and centre. (Harry)

Sometimes we have to wait a long time, but you know it can't be helped. (Henry)

It's just generally the issue of they have had to wait a long time […] You've got to think also there are diabetics among there that have to regulate their meals, and they have their set routine in terms of their meals and health. It's the same with everybody, but perhaps diabetics most, because it affects them straight away. I’ve known diabetics that have gone into a hypo [hypoglycaemia] because they were waiting around. Because they had a nine o'clock appointment and it got to eleven. It's just a real shame. (PH2)

There was a sense that the current model of care for nAMD would inevitably need to adapt to cope with this demand and consensus that monitoring in the community could reduce the clinical workload.

It will help shift a lot of the workload out of the hospital environment where they are overrun with this and putting it into a more capable environment with local optometrists. (Optom6)

[Monitoring in the community] will make less queues at the hospital. At the moment they're choc à bloc with people. (Ruth)

The optometrists, clinical advisors and commissioners also described how a shared care scheme represented an opportunity to enhance optometrists’ professional roles by developing their skills.

It's fantastic for the optometry profession, because it must give them much more exciting and interesting careers, and career progression, and variety within their work. (Comm3)

#### Potential for a more patient-centred model

Most health professionals believed that the current model of care was not appropriate for older patients with limited vision who had to regularly travel to the hospital. In line with this, the service users found it stressful travelling to and from the hospital for care. Those who used public transport had difficulty seeing bus numbers and often needed to get multiple buses. Others were driven by family or friends, but described the availability of parking as ‘awful’ (Arthur).

It’s not just getting to the hospital. It’s all that time afterwards, if you’ve got to get the bus, it’s—in the winter, it’s even worse. (Ralph)

One of the things that’s come out here is that everyone is, obviously, getting older. They’re stressed when they have to go out of the town because getting home when you’ve got … (Robert)

Oh, it's terrible. (Mandy)

So if they have someone in the town who is an optician and deals with us, it’s only a short distance from home. (Robert)

[Focus group extract]

The problem is that all the patients have got to go to the eye hospital all the time, which isn’t very patient-centric… It’s not very easy for people to get in once a month—which is obviously what Lucentis is about—for their assessment. Given that they are, almost by definition, elderly and with poor vision, it's not an ideal centre for it. (Comm2)

Monitoring in the community was described as a ‘wonderful’ idea (Elizabeth), particularly for those who lived further away from the hospital or older participants who had severe vision loss.

For me, living out of town in [small town], to get to an optician on the bus is easy, whereas it's a day's expedition to come into [city]. (Tracey)

Many rarely saw the same consultant or nurse at the hospital, and felt that staff were often impersonal as they were so busy. This was likened to ‘being on a conveyer belt’ (Pam).

If a doctor said, “Well, that's alright”, that's it. It's reassurance. I think they're trying to speed up time, and I know they're very busy and they obviously look at the photographs and they can see everything, but for patients’ feel-good interest, I always like a doctor…just to talk to you properly. (George)

The only criticism I would have is to try to find out how you're doing and whether you're getting worse or getting better, or stable, because they're all so busy. (Ruth)

These participants were, therefore, enthusiastic about the potential for continuity of care where they hoped to build up a relationship with their optometrist.

Many aspects of the eye hospital, it's so impersonal. I think that probably a system like you're suggesting would probably add a personal touch to it and a more one on one situation…That's the big thing, seeing the same person. Like I said, the personal touch…The relationship would build up. (Edward)

#### Perceptions of optometrists’ competency

The optometrists in the focus groups, who acknowledged that they had a special interest in nAMD, were very positive about the possibility of shared care and felt that their profession was more than capable of monitoring in the community. This was echoed by the commissioners, clinical advisors and public health representatives.

They're [optometrists] really incredible, impressive professionals, with just a huge amount of experience at looking at eyes. (Comm3)

However, several health professionals (from mixed professions) commented that ophthalmologists would resist shared care as they were not convinced of optometrists’ competence.

I think it’s the misconception that optometrists won’t do as good a job as secondary care. So I think that’ll be the biggest barrier. (Comm4)

This was considered to be problematic for shared care as there was uncertainty as to whether ophthalmologists would ever truly relinquish responsibility of patients.

Honestly, I think that clinicians aren’t always very good at letting go […] It will be an issue. (Comm3)

I would not want to close the door on them [stable patients], ever. (Ophthalm1)

There was hesitation by ophthalmologists as to whether optometrists were capable of monitoring nAMD. The ophthalmologists referred to how they frequently received incorrect referrals from optometrists. Furthermore, they highlighted the ways in which nAMD differed to other eye diseases where shared care schemes existed.

When you work in ophthalmology for quite some time you see just the amount of work that comes to you from inappropriate referrals. Really they're just doubling work up. (Ophthalm3)

This is not like glaucoma where you notice pressure or you don’t feel okay and refer back to the hospital. This is something…it's based on the scan and each patient is different. There's only a few parameters for glaucoma, whereas here there are…it’s complex […] So we can't expect an optometrist to…[laughter]. (Ophthalm5)

The ophthalmologists felt that the hospital provided an environment where they had access to all previous scans and other colleagues’ expertise, which enabled them to confidently make a clinical judgement. They expressed uncertainty about whether optometrists would have these resources. Ultimately, due to this perceived complexity of assessing the need for retreatment, and the support and resources available in the hospital, the ophthalmologists felt that monitoring patients in the community would be a compromise.

So what we are trying to say is hospital care is the best [laughter]. (Ophthalm6)

Several ophthalmologists felt that patients would prefer to remain being monitored by a consultant at the hospital, whom they would inherently trust. In line with this, service users with active nAMD also tended to be apprehensive about the level of optometrist competence in the community and commented that lengthy waiting times were secondary to receiving the best care for their condition.

If we put ourselves in their position what would we prefer to have? I'll prefer to be seen by a doctor in a hospital. (Ophthalm1)

So, you've got to have confidence in the person that is monitoring you […] I feel that to rely on somebody that has been trained up to identify problems can't really be as efficient as seeing an actual doctor who specialises in that subject, and because of that, I wouldn't be happy going to an optician. It might take you longer. We have sat up there for hours, but the end result is well worth it. (Henry)

The majority of service users described needing to be able to have faith in their optometrist if they were to participate in shared care. Those whose optometrist had diagnosed their nAMD commented that this gave them a sense of confidence in their optometrist's abilities.

I would trust my optician. He really seems to care. I would trust him. If my wet macular was stable, I'd be very happy to go to my optician because I've got confidence in him, because he detected it in the beginning. (Harriett)

However, a few service users were apprehensive about shared care and did not wholly trust the idea of monitoring by an optometrist. Those that expressed this did not have faith in their optometrist because the optometrist had not recognised the condition initially.

Personally I wouldn't have faith in the optometrist. I would much prefer to stay with the hospital. (Henry)

#### (Lack of) Communication between optometrists and ophthalmologists

Overall, the health professionals described the relationship between optometry and ophthalmology as poor.

Collaboration optometry and ophthalmology? No way. It's absolutely dreadful! [laughter] (Optom8)

These participants described how system-based issues accounted for the poor communication between the two professions. This was described by all professions except the ophthalmologists. For instance, most optometrists described how it could be extremely difficult to relay information to ophthalmologists because of incompatible email systems and variation in technology.

Our problem is we've got NHS.net in optometry and because everyone else, they're all NHS.co.uk. So their end isn't secure. We can do NHS.net to NHS.net, but we can't do NHS.net to NHS.co.uk, which is what all hospitals give their consultants. It's absolutely crazy. (Optom8)^i^

They've not embraced NHS.net at all. (Optom5)

Some optometric practices don't even have computers. Particularly in the [city] area where many of them are way behind. We actually had to buy them fax machines when this started to make it work. You'd expect most people had that sort of facility, but they didn't. To make it work, we would do that. So I think standardisation of forms across our units, across the country, and making those forms readily available, and everybody knows that they've got to look for the red-topped form in the practice or whatever, could possibly aid this model of shared care. (Optom2)

The other big issue is transfer. If you're actually going to transfer the data, they are massive, massive, massive files. I was talking to an optometrist who has an OCT and sends scans to an ophthalmologist, and he literally has to do it overnight. Just for one patient, it takes so long. (Optom1)

[Focus group extract]

All of the optometrists also commented that this had caused issues with their referrals to ophthalmologists in the past, in that they often got lost between the two professions. As a result, the optometrists stated that they would try to follow-up each referral by calling the consultant to ensure it had been received or they sent referrals via multiple technology methods to ensure that one would reach the consultant.

It does happen that patients will wander back in two weeks later and say, “Oh, you told me I'd be seen within a couple of weeks. I haven't heard a thing”. Then we contact the hospital and we think, “Okay, what's happening?” (Optom2)

Many participants (health professionals and service users) explained that one of the key concerns of nAMD was the rapid progression of the disease if the condition became active. In particular, several service users described the devastating consequences of their vision deteriorating within days.

Last week I was all-seeing and driving and everything, and on Thursday I thought, “There's something missing on that signpost”, and the glare was terrible. So then I couldn't read the paper at lunchtime…To me, it's been a disastrous week. I can no longer drive. I can no longer read. This is a week! To me, that's a disaster. It's very frightening. (Pat)

Therefore, the participants, particularly the ophthalmologists and service users, expressed concerns about a potential delay between primary and secondary care sectors if retreatment was required.

The problem is that the more steps you have in the system, the longer it takes. We don't have the time. (Edward)

If you are in the community and it’s not stable, then you have to have a very quick way of getting back to treatment. So those are my concerns. (PH1)

The health professionals emphasised the importance of the two professions working collaboratively so that an efficient pathway could be developed.

You'd need to be sure that there’s a seamless process between community and hospital, and that nothing drops through the cracks. So I think they would need to make sure that there’s a robust recall service, and that if there is an issue, that there’s a pathway back for the patient into secondary care. (Comm2)

#### The cost of shared care

Although the optometrists and the ophthalmologists believed that financial considerations should be secondary to patient outcomes, the other health professionals stated that a harsh reality of healthcare was that shared care would not be commissioned unless it ‘got the most out of the NHS pound’ (PH2).

You've got to show the CCGs that you’re saving money over sending them into the hospital. Because otherwise, they’re just not going to commission it. (PH1)

The commissioner participants undertook several roles professionally and were often optometrists or GPs alongside this position. The multiple perspectives from a commissioning and clinical point of view appeared to be in conflict when contemplating nAMD care, in terms of patient outcomes and financial efficiency.

Patients would like it [being monitored in the community] because it's much closer to home, they don't have to go to hospital. They don't have to sit and queue and wait in pain, park and all that sort of stuff that patients usually tell you…[…] From a commissioner's perspective… In terms of saving and shifting costs across the health system for eye care services, it certainly doesn't achieve that. (Comm4)

Among the health professionals, there was disagreement as to whether shared care would represent a cost-efficient model. For instance, many perceived that differential fees for optometrists and ophthalmologists, as well as decreased costs of managing sight loss, may be financial incentives to commission shared care.

It would be a cost efficient option for the commissioners because we would be paying something like £60 for an optometrist to measure the patient's visual acuity, rather than £100 and whatever it is for a consultant outpatient episode. (Comm4)

You could use the monitoring to stop the wet AMD getting worse, so that it's kind of preventative, then you would be addressing the public health indicator of dropping those numbers of people registered with AMD sight loss. (PH2)

However, there was agreement that the equipment to obtain OCT images, although considered essential for monitoring nAMD, was an expensive piece of kit that not all practices might be able to invest in. The clinical advisors and public health representatives felt that CCGs should provide OCTs, although most of the commissioners and the ophthalmologists felt it should be self-funded to demonstrate a level of commitment to monitoring in the community.

Well, it's on the optometrists, really. Whether they've got the kit or whether they will want to invest in one. I think what it will be is that you'd have a small group of practices within a particular area, who will show keenness. […] They will be the more cutting edge practices. (Comm6)

I think CCGs should pay for that [an OCT], the NHS. I don't think it should be the optometrist. (PH2)

While the ophthalmologists stated that they took around 15 min to see a patient and determine the need for retreatment, the optometrists’ estimates varied between 20 and 40 min, with the latter estimate of up to 40 min being more realistic to make a clinical decision and explain the results to the patient.

I'll allow 30 and probably spend 40 […] But once you've got an OCT sitting there and you start looking at it, and you really want to explain it to the patient and they say, “Oh, thank you. No one ever tells me anything like that at the hospital. It makes you feel so good at the end. You've actually told the patient what's really wrong and, “Rest assured my dear, it's not getting any worse, so we don't need to send you back. ‘‘Oh, thank you!” It probably would take me 40 minutes. (Optom8)

Several commissioners and one clinical advisor also highlighted a potential conflict between practices’ clinical and commercial interests, and stated that optometrists would need to be paid a sufficient amount to ensure that shared care was financially possible.

They [optometrists] make the majority of their money on spectacles, and things like that. They get paid a minimal amount for seeing patients… They need to be paid at a certain level, which allows them to invest in the equipment, and IT systems for regular follow up, all that kind of stuff. (Comm2)

There were also concerns that CCGs might be charged for repeat tests if ophthalmologists lacked trust in optometrists’ judgements.

We would want to make sure we would only be being charged for that element of it, and they [ophthalmologists] wouldn't go on and repeat all the tests again. (Comm1)

#### The importance of specialist training

The health professionals spent a considerable amount of time discussing how they felt training should be delivered, in terms of which method was most effective for learning, and ensuring the training was delivered in a way that would reassure ophthalmologists that optometrists were being trained to a high standard.

“I think the training needs to be very carefully designed”. (PH2)

“The ophthalmologists have to believe in the competence of the optometrist. It's important that they have belief in the quality of the accreditation”. (Optom4)

Virtual training was deemed appropriate for providing a foundation level of knowledge, although most felt that this alone would not be sufficient to train the optometrists. In particular, the ophthalmologists were unconvinced by the applicability of a virtual trial.

I'm sure in your studies you will find 100% virtual case studies that you give, that you will find good correlation between what the ophthalmologist would say and what the optician would say in a virtual case. You can't just say “Yes, the optom has 100% exactly the same as the ophthalmologist, which says that now they are just as good.” (Ophthalm3)

Clinical experience—whereby optometrists would gain experience of monitoring nAMD patients in a hospital setting—was viewed as an essential component of training.

I would suggest that they [optometrists] would spend a certain amount of time in a consultant clinic… That will help the consultants gain a bit of confidence in the optom as well. So it’s a working partnership going on there. (Comm2)

I think they need to come and see the real patient. There's no point on sitting on the MediSoft or whatever and clicking boxes and thinking they know it all. Real life is not like that. I mean there are the OCT scans like that, sometimes they can be very devious and very challenging and very confusing… (Ophthalm2)

The majority of participants felt that having ophthalmologists’ supervising this clinical training would provide assurance about the optometrists’ competence and enable greater collaboration between the two professions. However, they acknowledged that this would be time-consuming.

I think they would be involved if they had an element of control over it. If they'd done the training and if they knew who they were sending the patients out to and they knew what protocols were being followed, the service spec. You don't get these things to work unless you've got clinical buy in. You just don't. You can set up all you want, but you've got to get the clinicians involved. (Optom5)

Obviously it has to be somebody senior who has responsibility of training them and signing them off. (Ophthalm1)

Is that something that you would be willing to do, to train an optometrist? (DT)

Where's the time? (Ophthalm1)

Exactly. (Ophthalm4)

We'd have to stop doing the MD clinics and start training. (Ophthalm2)

[Focus group extract]

## Discussion

This study has provided important insights into health professionals’ and service users’ perspectives of shared care for patients with nAMD. Overall, the findings suggest that the needs and preferences of those with nAMD appear to be at odds with the current service being provided. There was enthusiasm from health professionals and service users for a shared model of care as it was felt to have the potential to relieve HES burden and represent a more patient-centred option. However, there was uncertainty as to how this could operate in real life, including uncertainty about start-up costs and payment scales, about demand and patient flows, and about how hospital-community communications would work in practice. This suggests the need for pilot studies to work out the practicalities and provide real-life proof of concept.

There appeared to be unmet needs for those with nAMD that could be better met by delivery of a shared hospital and community care scheme. In line with previous research, the service users described frustration at the lack of support and information they received at hospital^14^ ^15^ and felt that being monitored in the community would enable them to build up a relationship with an optometrist. Furthermore, a shared care scheme was attractive due to the convenience of being monitored closer to their homes, as found in other studies.^7^ ^12^ With the burden of hospital follow-up visits for patients with stable nAMD,^6^ the health professionals felt that shared care could also relieve the ophthalmology workload.

However, a number of possible barriers to implementing a shared care scheme were identified. Several ophthalmologists and service users voiced concerns about the optometrist's competence and a potential delay in retreatment should the need arise. Previous research has found that patients decline other shared care schemes because of the reputation of and familiarity with hospital services,^12^ and specialists are not convinced of optometrists’ expertise, even with additional training.^16^ O'Connor *et al*^12^ conducted interviews with optometrists, ophthalmologists and patients who had participated in a shared care scheme for dry AMD, diabetic retinopathy and glaucoma. They concluded that without time invested in relationship building, ophthalmologists’ reservations about the capabilities of optometrists could be a barrier to implementing shared care. In the current study, the health professionals felt training for optometrists, under the supervision of ophthalmologists, would improve the communication and trust between the two professions that shared care would require.

The health professionals considered the financial implications of moving to a shared care model. There was agreement that optometry practices may struggle to obtain appropriate equipment, but uncertainty as to how funding for OCTs would be provided. Studies exploring optometrists’ perspectives of extending or enhancing their roles have highlighted a conflict between the retail and clinical side of the optometric practice.^10^ ^11^ ^16^ Although it is important to note that the points raised are an interpretation of the cost of shared care, rather than an objective measure, there was uncertainty as to whether shared care was financially more efficient than the current model of care. However, Amoaku *et al*^6^ state that adapting nAMD care may be a necessary investment to improve patient care, increase productivity by maximising the use of experienced clinicians, and ultimately reduce the health and social costs of sight loss in the long term.

### Strengths and limitations

The main strength of this research is the use of qualitative methodology to provide important insights into the under-researched area of whether shared care for nAMD can and should be implemented by assessing its acceptability to service users and healthcare professionals.^17^ Focus groups provided an opportunity for participants to prompt each other about a range of issues relating to shared care which may not have been considered individually. Interviews provided a rich account of the perceived feasibility and acceptability of shared care scheme as the findings from the focus groups could be followed up further and explored in-depth.

Participating optometrists and ophthalmologists were attending specialist conferences; so they may represent a more motivated and enthusiastic group of professionals. Similarly, service users were recruited from Macular Society support meetings; so they may represent a more proactive and informed group of patients.^15^ However, a purposeful sampling approach was adopted to ensure that we captured a range of perspectives from different participants within these constraints. Furthermore, positive and negative views were provided, suggesting participants carefully considered the practicalities of implementing shared care. Although service users attended the same eye hospital for their nAMD care, the themes relating to current experiences of hospital care have also been highlighted in previous research.^7^ ^12^ ^15^

None of the participants had the experience of shared care for nAMD. It has been questioned whether evaluations of hypothetical scenarios accurately relate to judgements in real-life situations.^18^ However, the issues identified in this study mirror many of the findings from other studies in which participants had experience of shared care for other eye conditions. Our findings highlighted similar potential barriers which could limit the feasibility of a shared care scheme, including perceptions of optometrists’ competence, poor interprofessional communication, and the cost of implementing shared care.^11^ ^12^

## Conclusion

This study demonstrates that there is enthusiasm for shared care for monitoring nAMD as this could better meet the needs of those with the condition than the current model of care. However, ophthalmologists and service users would need reassurance that greater convenience would not be substituted by a lower standard of eye care, both in terms of optometrist competence and the speed of the referral pathways back into secondary care if retreatment was deemed to be necessary. There also seemed to be poor communication and trust between ophthalmologists and optometrists; most professions agreed that, if shared care were to be implemented, it would need to be done in a way that ensures the two professions were working more collaboratively. These findings would need to be incorporated into the planning of any pilot scheme for shared care of nAMD.

## Footnotes

i NHS.net and NHS.co.uk are email systems for NHS employees in England and Scotland.

## References

[1] Minassian DC, Reidy A, Lightstone A Modelling the prevalence of age-related macular degeneration (2010–2020) in the UK: expected impact of anti-vascular endothelial growth factor (VEGF) therapy. Br J Ophthalmol 2011;95: 1433–6. 10.1136/bjo.2010.195370 21317425

[2] Bunce C, Xing W, Wormald R Causes of blind and partial sight certifications in England and Wales: April 2007–March 2008. Eye 2010;24:1692–9. 10.1038/eye.2010.122 20847749

[3] RNIB. Future sight loss UK (1): The economic impact of partial sight and blindness in the UK adult population executive summary. RNIB, 2013 http://www.rnib.org.uk/sites/default/files/FSUK_Summary_1.pdf (accessed 7 Oct 2014)

[4] Bennion AE, Shaw RL, Gibson JM What do we know about the experience of age-related macular degeneration? A systematic review and meta-synthesis of qualitative research. Soc Sci Med 2012;75:976–85. 10.1016/j.socscimed.2012.04.023 22709445

[5] National Institute for Health and Clinical Excellence. Ranibizumab and pegaptanib for the treatment of age-related macular degeneration: NICE technology appraisal guidance 155, London, 2008.

[6] Amoaku W, Blakeney S, Freeman M Optimising patient management: act now to ensure current and continual delivery of best possible patient care. Eye 2012;26 (Suppl 1):S2–21. 10.1038/eye.2011.343 22302094 PMC3292344

[7] Bhargava JS, Bhan-Bhargava A, Foss AJ Views of glaucoma patients on provision of follow-up care; an assessment of patient preferences by conjoint analysis. Br J Ophthalmol 2008;92: 1601–5. 10.1136/bjo.2008.140483 18664502

[8] Gray SF, Spry PG, Brookes ST The Bristol shared care glaucoma study: outcome at follow up at 2 years. Br J Ophthalmol 2000;84:456–63. 10.1136/bjo.84.5.456 10781507 PMC1723467

[9] Gray SF, Spencer IC, Spry PG The Bristol shared care glaucoma study-validity of measurement and patient satisfaction. J Public Health Med 1997;19:431–6. 10.1093/oxfordjournals.pubmed.a024673 9467150

[10] Myint J, Edgar DF, Kotecha A Barriers perceived by UK-based community optometrists to the detection of primary open angle glaucoma. Ophthal Physl Opt 2010;30:847–53. 10.1111/j.1475-1313.2010.00792.x 21205271

[11] Konstantakopoulou E, Harper RA, Edgar DF A qualitative study of stakeholder views regarding participation in locally commissioned enhanced optometric services. BMJ Open 2014;4:e00478 10.1136/bmjopen-2013-004781 PMC4039782 24875489

[12] O'Connor PM, Harper CA, Brunton CL Shared care for chronic eye diseases: perspectives of ophthalmologists, optometrists and patients. Med J Aust 2012;196:646–50. 10.5694/mja11.10856 22676881

[13] Strauss A, Corbin J. *Basics of qualitative research: techniques and procedures for developing grounded theory*. 2nd edn. London: Sage, 1998:57–71.

[14] Burton AE, Shaw RL, Gibson JM “I'd like to know what causes it, you know, anything I've done?” Are we meeting the information and support needs of patients with macular degeneration? A qualitative study. BMJ Open 2013;3:e003306 10.1136/bmjopen-2013-003306 PMC3822314 24202055

[15] Mitchell J, Bradley P, Anderson SJ Perceived quality of health care in macular disease: a survey of members of the Macular Disease Society. Br J Ophthalmol 2002;86:777–81. 10.1136/bjo.86.7.777 12084749 PMC1771190

[16] Holtzer-Goor KM, Plochg T, Lemij H Why a successful task substutition in glaucoma care could not be transferred from a hospital setting to a primary care setting: a qualitative study. Implement Sci 2013;8:14 10.1186/1748-5908-8-14 23351180 PMC3576268

[17] Rapport F, Storey M, Porter A Qualitative research within trials: developing a standard operating procedure for a clinical trials unit. Trials 2013;14:54 10.1186/1745-6215-14-54 23433341 PMC3599333

[18] Collette JL, Childs E Minding the gap: meaning, affect, and the potential shortcomings of vignettes. Soc Sci Res 2011;40: 513–22. 10.1016/j.ssresearch.2010.08.008

